# Development of a New Dry Powder Aerosol Synthetic Lung Surfactant Product for Neonatal Respiratory Distress Syndrome (RDS) – Part I: *In Vitro* Testing and Characterization

**DOI:** 10.1007/s11095-024-03740-z

**Published:** 2024-08-07

**Authors:** Mohammad A. M. Momin, Dale Farkas, Michael Hindle, Felicia Hall, Robert M DiBlasi, Worth Longest

**Affiliations:** 1 Department of Pharmaceutics, Virginia Commonwealth University, Richmond, Virginia USA; 2 Department of Mechanical and Nuclear Engineering, Virginia Commonwealth University, 401 West Main Street, P.O. Box 843015, Richmond, Virginia 23284-3015 USA; 3 Center for Respiratory Biology and Therapeutics, Seattle Children’s Research Institute, Seattle, Washington USA

**Keywords:** infant aerosol therapy, surfactant aerosol, surfactant replacement therapy, synthetic lung surfactant, respiratory distress syndrome

## Abstract

**Purpose:**

Improving the deep lung delivery of aerosol surfactant therapy (AST) with a dry powder formulation may enable significant reductions in dose while providing improved efficacy. The objective of Part I of this two-part study was to present the development of a new dry powder aerosol synthetic lung surfactant (SLS) product and to characterize performance based on aerosol formation and realistic *in vitro* airway testing leading to aerosol delivery recommendations for subsequent *in vivo* animal model experiments.

**Methods:**

A new micrometer-sized SLS excipient enhanced growth (EEG) dry powder formulation was produced via spray drying and aerosolized using a positive-pressure air-jet dry powder inhaler (DPI) intended for aerosol delivery directly to intubated infants with respiratory distress syndrome (RDS) or infant-size test animals.

**Results:**

The best-case design (D2) of the air-jet DPI was capable of high emitted dose (> 80% of loaded) and formed a < 2 µm mass median aerodynamic diameter (MMAD) aerosol, but was limited to ≤ 20 mg mass loadings. Testing with a realistic *in vitro* rabbit model indicated that over half of the loaded dose could penetrate into the lower lung regions. Using the characterization data, a dose delivery protocol was designed in which a 60 mg total loaded dose would be administered and deliver an approximate lung dose of 14.7–17.7 mg phospholipids/kg with a total aerosol delivery period < 5 min.

**Conclusions:**

A high-efficiency aerosol SLS product was designed and tested that may enable an order of magnitude reduction in administered phospholipid dose, and provide rapid aerosol administration to infants with RDS.

## Introduction

Aerosol surfactant therapy (AST) has been envisioned for a number of decades as a method to effectively treat infant respiratory distress syndrome (RDS) without the side effects and risks associated with surfactant liquid instillation through an endotracheal tube and other less invasive liquid bolus installation techniques [1–6]. All commonly used surfactant administration techniques (i.e. endotracheal tube (ETT) delivery; INSURE, LISA, MIST [7–9]) require intubation beyond the vocal cords with a tube or catheter and instillation of large liquid volumes (1.2–4 mL/kg) directly to the lungs [2, 9–12]. Side effects of this invasive approach often include significant airway obstruction from surfactant reflux, oxygen desaturation (frequently requiring resuscitation), bradycardia, pneumothorax, hemodynamic instability, reduced cerebral blood flow [2, 11, 13–15], and the relatively high risks associated with infant intubation (e.g., > 50% severe oxygen desaturations) [16, 17]. Other limitations of surfactant liquid bolus instillation include highly heterogeneous distribution of surfactant within the lungs, surfactant leakage, and frequent alveolar flooding [18–20], as well as structural damage to the alveolar network and increased inflammation of the lungs [21–23]. In contrast, successful AST is envisioned to improve treatment outcomes through a number of mechanisms, including improved transport to the alveolar region, reduction of risks and side effects associated with liquid bolus instillation, and less alveolar flooding and associated stress [1, 2]. In addition, an effective AST product could be delivered during non-invasive ventilation (NIV) [4, 24, 25], preventing the need for intubation in many cases, and could also be administered in low resource settings where intubation, mechanical ventilation and liquid bolus instillation are not possible [26].

Animal model studies over multiple decades have demonstrated the effective use of AST for infant RDS-like conditions using both liquid nebulized [5, 22, 23, 25, 27–29] and dry powder aerosols [21, 30–32]. Considering human subject testing with liquid nebulized aerosols, six surfactant aerosol trials have been conducted in preterm infants over the last five years with the primary outcome of NIV failure and progression to invasive mechanical ventilation. Results of these clinical studies have varied between significant reduction in NIV failure rate [6, 24, 33], success only in older age groups or non-significant trends [34, 35], and no improvement [36]. Dry powder AST has shown promising results in animal models [21, 30–32], but has not progressed to human subjects testing in recent years, despite promising preliminary results in a small human subject trial over four decades ago [3].

While animal models and human subject trials have demonstrated the potential benefits of AST, current aerosol delivery techniques are associated with several limitations that have impacted performance to varying degrees. First, delivered doses are often required to be much larger than the amount of surfactant expected for biological efficacy. It is estimated that approximately 10 mg of phospholipids (PL; primarily DPPC) per kg of body weight should have a significant impact on alveolar surface tension [37–40]. In human subject studies with nebulized surfactants, the typical range of nebulized dose is 100–400 mg PL/kg, with successful studies typically implementing up to two [24] or four [35] doses. These nebulizer doses are equivalent to or larger than current clinical liquid instilled doses of 100–200 mg PL/kg [10, 41]. Secondly, nebulized aerosol delivery typically takes much longer than liquid bolus instillation, due to poor lung delivery efficiency, with nebulization times requiring ~ 30 min to 2.5 h [5, 24]. Delivery times in animal models with dry powder aerosols have been improved, with multiple capsules delivered over an ~ 15 min period, with a device requiring up to ~ 400 actuations [32]. Thirdly, we expect poor lung delivery efficiency and poor alveolar delivery efficiency regularly occur resulting in highly heterogeneous surfactant distributions in the alveolar region. This observation is supported by gamma scintigraphy studies in which a vast majority of the nebulized aerosol liquid (e.g., 100-fold more) is found in the dependent (gravity-down) lung of side-lying animals [18, 25, 42]. Finally, high extrathoracic and upper tracheobronchial airway deposition of aerosol within existing systems leads to persistent side effects that are also common with liquid bolus instillation including surfactant reflux, oxygen desaturation and a need for infant resuscitation [24, 36].

We propose that the efficacy of AST can be increased at reduced doses, with associated reduced cost, if highly active surfactant formulations can be rapidly and effectively delivered to the lungs, beyond the lobar bronchi and preferentially all the way to the alveolar region with a relatively homogeneous deposition pattern. Our group is developing a new dry powder aerosol synthetic lung surfactant (SLS) product with the goal of rapid and efficient aerosol delivery to the alveolar region that can be applied during invasive mechanical ventilation, NIV support, or directly to an infant using the nose-to-lung route. Elements of this new product include an excipient enhanced growth (EEG) aerosol delivery strategy [43, 44], highly dispersible micrometer-sized surfactant formulation [45] and high efficiency infant dry powder aerosol delivery system (iDP-ADS) [46, 47]. As a core technology of this product, the EEG targeting strategy involves beginning with a micrometer-sized particle dry powder aerosol that can efficiently penetrate the delivery system, infant (or test animal) extrathoracic airways and upper lung regions [46, 48]. Hygroscopic growth excipients (mannitol and sodium chloride) included in the formulation cause the micrometer-sized aerosol to increase in size during transport into the lungs through condensational growth and help foster alveolar deposition as opposed to the dose exhalation that would otherwise occur [49, 50]. A balance is often required with an initial aerosol size that is just small enough for effective lung entry but that also has sufficient size and growth potential (in the form of excipient content) [51] to achieve deep lung deposition. With high dose applications, it is also important to control the total mass and delivery rate of the powder and excipients to the lungs [52].

A key component of the new AST product is the dry powder formulation consisting of pharmaceutical-grade phospholipids (DPPC and POPG), a synthetic surfactant protein mimic (B-YL), hygroscopic excipients (MN and NaCl) and a dispersion enhancer (l-leucine). Compared with nebulized liquids, potential advantages of the dry powder formulation include the formation of a smaller-particle aerosol with less input energy, more rapid delivery, improved stability and the ability to better integrate delivery flow pathways with existing ventilation equipment. Based on previous work with an animal-derived surfactant product (Survanta®, Abbvie, North Chicago, IL), a spray-dried Survanta-EEG formulation was shown to have very high efficacy at a PL dose of ~ 1.5 mg/kg in a rat surfactant washout model [21]. Spray drying of the Survanta-EEG formulation was optimized and performed using a vibrating-mesh-based system [45], and aerosol formation was achieved with a custom small-animal DPI device [53]. Considering synthetic lung surfactants, third-generation synthetic surfactant protein mimics, such as Mini-B, Super Mini-B and B-YL, have shown high potential for surface tension reduction and efficacy when combined with pharmaceutical-grade PLs [4, 29, 54–56]. Walther and coworkers have extensively developed the SP-B protein mimic B-YL and shown animal model efficacy with dry powder formulations in multiple animal species, with required doses ranging from 100—240 mg of powder / kg delivered once or twice, depending on biological response [32, 55, 57]. It is proposed that a SLS-EEG formulation containing B-YL combined with a high efficiency aerosolization device can provide improved surfactant efficacy at lower doses.

Components of the iDP-ADS include a positive pressure gas source, aerosolization engine, pressure monitoring and control (PMC) unit, diffusional flow pathway and a patient interface. Considering the aerosolization engine, our group has extensively developed the air-jet DPI concept [46, 47, 58–63], which enables high efficiency aerosol formation across a range of available air volumes. The air-jet DPI consists of small diameter inlet flow passages, an aerosolization chamber, an outlet flow passage (capillary) and a diffusional flow pathway [61]. The design of these relatively simple elements can be engineered to control the amount of aerosol deaggregation, emitted dose (ED) and the formulation delivery rate from the device. For administering the SLS product to an infant-size rabbit surfactant washout model (e.g., 1.2–1.6 kg [28]), an air-jet DPI is needed that can accommodate 10–30 mg powder loading followed by efficient aerosol formation with low air volumes over multiple actuations. A new PMC unit is also needed for aerosol delivery to sedated animals as well as a simple endotracheal tube interface.

An additional component of the delivery strategy to be accomplished with the iDP-ADS is to administer the aerosol with positive-pressure breaths. For both ETT and future nose-to-lung administration of the aerosol, the delivery interface will form an airtight seal with the infant’s airways to facilitate positive-pressure breaths and exhalation will be controlled through the PMC unit. Positive-pressure administration of the surfactant aerosol is expected to open (recruit) collapsed lung regions in the treatment of RDS, and the subsequent deposition of surfactant with rapid formation of a surface lining layer will maintain the patency of these regions, preventing the inflammation and injury that arise from cyclic opening and collapse of alveoli [1, 2, 64]. Based on ventilation of preterm neonates, targeted maximum and minimum delivered pressures will be 25 ± 2 to 5 ± 2 cm H_2_O [65–68]. A method is currently needed to achieve these targeted pressures during aerosol delivery with the iDP-ADS across a range of potential lung compliances.

The overall goal of this two-part study is to present and characterize a new dry powder aerosol SLS product including an EEG aerosol delivery strategy for targeting aerosol deposition to the alveolar region, a micrometer-sized SLS-EEG dry powder formulation, and a delivery system (iDP-ADS) for the administration of the dry powder aerosol to the lungs of intubated infants or similarly sized test animals. In Part I of this study, we focus on development of the iDP-ADS and formulation, as well as *in vitro* characterization. In Part II we report *in vivo* efficacy data using an infant-sized rabbit model of neonatal RDS. Specifically, the objective of Part I of this two-part study is to present the development of the new aerosol SLS product, including details about the initial aerosol delivery strategy, formulation and iDP-ADS, and to characterize performance based on aerosol formation and realistic *in vitro* airway testing leading to aerosol delivery recommendations (i.e., a complete dose delivery protocol) for the subsequent *in vivo* animal model testing. The SLS-EEG formulation was produced using spray drying techniques with a mesh-based nebulizer system similar to techniques developed for Survanta-EEG [45]. A new air-jet DPI was developed to efficiently aerosolize relatively large powder masses with limited air volumes for aerosol delivery to sedated animals, and included comparison of two designs (D1 and D2). Our challenge was to develop a formulation and delivery system capable of generating a micrometer-sized surfactant powder aerosol using ~ 10 mL of dispersion air delivered at a flow of ≤ 3 L/min over a time interval of < 300 ms. Initial *in vitro* testing was performed to characterize the SLS-EEG formulation primary particle size, test aerosol formation characteristics, select a leading air-jet DPI, explore an aerosol dose delivery strategy and evaluate expected lung dose and lung pressure profiles using a new *in vitro* airway model. Outcomes of this Part I study including a best-case device, SLS-EEG formulation and dose delivery protocol were then advanced to Part II for *in vivo* efficacy testing using an intubated rabbit surfactant-washout model of neonatal RDS.

## Materials and Methods

### Formulation Materials

Phospholipids (dipalmitoyl phosphatidylcholine (DPPC) and 1-palmitoyl-2-oleoyl-sn-glycero-3-phospho-(1'-rac-glycerol) (sodium salt) (POPG)) and surfactant protein mimic peptide (B-YL) were purchased from Avanti Polar Lipids, Inc. (Alabaster, AL) and CSBio (Menlo Park, CA), respectively. L-leucine was purchased from Sigma-Aldrich Chemical Co. (St Louis, MO). Sodium chloride and D-mannitol together with other chemicals were purchased from Fisher Scientific Co. (Hanover Park, IL). Throughout the study, freshly collected deionized water was used.

### Formulation Production

Multiple batches of Nano spray-dried (SD) SLS-EEG powder formulation were prepared by spray drying of feed dispersions containing phospholipids (DPPC and POPG), surfactant protein mimic (B-YL), hygroscopic excipients (mannitol and sodium chloride) and dispersion enhancer (l-leucine) using the Buchi Nano Spray Dryer B-90 HP (Büchi Labortechnik AG, Flawil, Switzerland) following the same procedure as previously reported [45]. Briefly, the feed dispersions were prepared with a solids composition of DPPC 52.5%, POPG 7.5%, B-YL 5%, mannitol 9%, sodium chloride 6% and l-leucine 20% w/w in an ethanol–water (5:95% v/v) co-solvent system, which was sonicated for 40 min in a heated water bath (45–55°C) with intermittent manual shaking every 10 min. The total solids content of the feed dispersion was 0.125% w/v. The feed dispersion was spray-dried using the small nozzle option at a spray rate of ~ 0.40 mL/min. Throughout the spray drying process, the inlet temperature was set to 70 °C, resulting in an outlet temperature of 35–38 °C. During spray drying, the feed dispersion was continuously stirred using a magnetic stirrer as excess feed dispersion was recycled into the stock until completion of the batch processing. The Nano SD SLS-EEG powders were collected on an electrostatic precipitator and transferred into glass vials and stored in a desiccator (0% RH) at -20 °C. It is noted that while we are targeting ~ 1 µm primary particle aerodynamic diameters, the formulation nomenclature of Nano is used to denote the spray drying system (Buchi Nano Spray Dryer B-90 HP) implemented for production.

### Analytical Methods: DPPC and B-YL HPLC Methods

Validated HPLC methods were used to measure the content uniformity of DPPC and B-YL in the SLS-EEG formulations together with quantifying the aerosol performance by measuring DPPC deposition. For DPPC, the system consisted of an Alliance 2695 Separation Module with a photodiode array UV detector and Empower 3 data acquisition software (Waters Corporation, Milford, MA). The sample injection volume was 100 µL and column temperature was 45ºC. The chromatographic separation was achieved using the Waters XSelect CSH C18 XP, (2.5 μm, 4.6 × 100 mm; Waters Corporation, Milford, MA). A gradient elution system employed: Solvent A: 5 mM tetrabutylammonium bisulfate in water, pH 8.52: Solvent B acetonitrile: isopropanol: ammonium hydroxide: perchloric acid (40:60:0.05:0.0005 v/v), and Solvent C: acetonitrile, pumped at a flow rate of 0.8 mL/min. Following chromatographic separation UV absorbance was measured at 204 nm. The retention time for DPPC was ~ 9 min. For B-YL, the system was modified to employ a gradient elution with, Solvent A: water:trifluoroacetic acid (100:0.075 v/v), Solvent B: acetonitrile: trifluroacetic acid: polysorbate 80 (100:0.075:0.002 v/v), an injection volume of 40 µL and a detection wavelength of 214 nm. All other conditions were unchanged. A DPPC stock solution (2 mg/mL) was prepared by dissolving a sufficient amount of DPPC in 2.5 mM TBA in water: ethanol: polysorbate 80 (15:85:0.005 v/v (diluent)). Diluted standard solutions of DPPC in the concentration range of 50 – 400 µg/mL were prepared by further dilution of the stock standard solution in diluent. A B-YL stock solution (100 µg/mL) was prepared by dissolving a sufficient amount of B-YL in diluent. Diluted standard solutions of B-YL in the concentration range of 7 – 25 µg/mL were prepared by further dilution of the stock standard solution in diluent.

### Formulation Characterization: DPPC Content Uniformity

The DPPC content of the powders was evaluated by quantitative analysis of the DPPC using the HPLC–UV method. Approximately 1 mg of Nano SD SLS-EEG formulation was dissolved in 3 mL diluent and quantitatively analyzed for the DPPC content. Triplicate samples were prepared and analyzed for each of the powder samples.

### Formulation Characterization: B-YL Content Uniformity

The B-YL content of the powders was evaluated by quantitative analysis of the B-YL using the HPLC–UV method. Approximately 1 mg of Nano SD SLS-EEG formulation was dissolved in 3 mL diluent and quantitatively analyzed for the BY-L content. Triplicate samples were prepared and analyzed for each of the powder samples.

### Formulation Characterization: Laser Diffraction Particle Sizing

Powder dispersibility and primary particle size of the Nano SD SLS-EEG formulation was determined using laser diffraction at multiple dispersion pressures. Specifically, components employed included the Sympatec HELOS (Sympatec GmbH, Germany) equipped with a RODOS/M disperser and an ASPIROS sample feeder. A small glass vial was filled with approximately 3 mg of spray-dried powder, placed in the sample feeder and dispersed in the laser beam using the RODOS/M disperser with an R1 lens at dispersion pressures of 0.5 and 4.0 bar. The size distribution results (Dv_10_, Dv_50_ and Dv_90_) were obtained using Sympatec WINDOX 5.10 software (Sympatec GmbH, Germany). Size distribution results determined at 4 bar are assumed to be close to fully deaggregated primary particles, and the difference between the size distributions at 0.5 and 4 bar provides an indication of the powder dispersibility.

### Formulation Characterization: Scanning Electron Microscopy

The particle morphology of the Nano SD SLS-EEG formulation was evaluated using a Zeiss EVO-50XVP scanning electron microscope (SEM) (Carl Zeiss, Oberkochen, Germany) at an accelerating voltage of 5.0 kV. A small amount of powder sample was mounted on a metal SEM stub using double-sided adhesive tape and loose powders were removed using compressed air. The samples were sputter coated with gold grain using an EMS550X sputter coater (Electron Microscopy Sciences, Hatfield, PA) before taking the images.

### Formulation Characterization: X-ray Powder Diffraction (XRPD)

The Nano SD SLS-EEG formulation was characterized using the Rigaku Miniflex 6G (Rigaku, Japan) X-ray powder diffraction technique. Briefly, a small amount of powder was placed on the center of the XRPD sample holder and then the powder surface was gently smoothed using a glass slide. The samples were scanned using a continuous mode in the range of 3–50° 2θ at a scan speed of 1.5°/min.

### Formulation Characterization: Moisture Sorption

To understand the water uptake of the Nano SD SLS-EEG formulation as a function of relative humidity (RH), samples were exposed using the dynamic vapor sorption system (DVS Adventure, Surface Measurement Systems Ltd., UK). Briefly, a small amount of powder sample (about 5 mg) was equilibrated at 0% RH and then exposed to increasing RH from 0 to 95% (sorption cycle) and decreasing RH from 95 to 0% (desorption cycle). At each RH, once the defined equilibrium condition (the rate of mass change (dm/dt) less than 0.002%) was achieved, the RH was changed to the next level.

### Development of the iDP-ADS

**Fig. 1:**
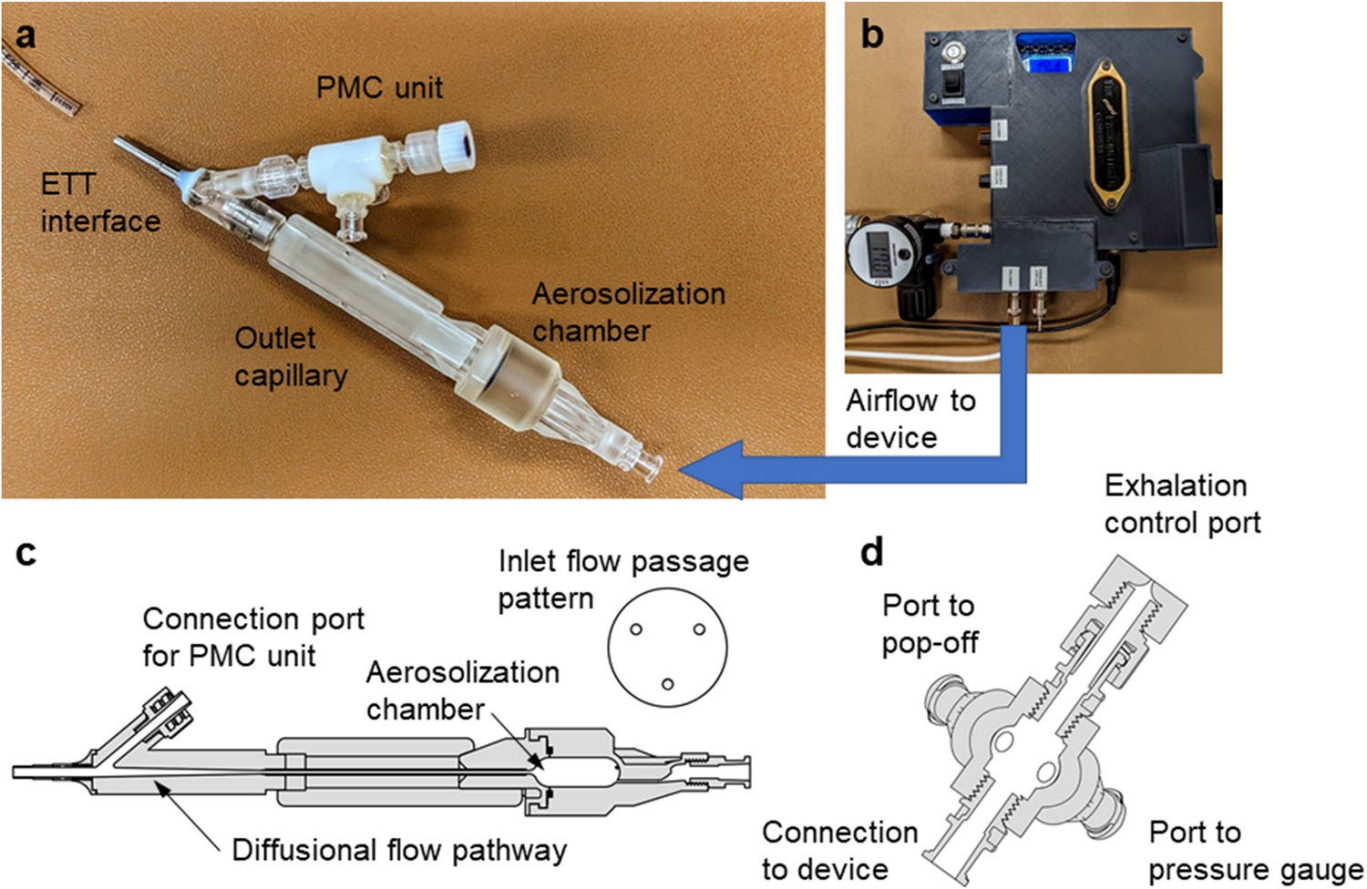
An overview of the infant dry powder aerosol delivery system (iDP-ADS) including the (**a**) prototyped D2 air-jet DPI with key components labeled, (**b**) electromechanically controlled timer air source (EM Timer), (**c**) internal flow pathway (D1 device) with key components labeled, and (**d**) the pressure monitoring and control (PMC) unit.

An overview of the iDP-ADS is provided in Fig. 1, which illustrates the main elements including the electromechanically controlled timer air source (EM Timer), aerosolization engine, diffusional flow pathway, PMC unit, and interface for direct connection with 2.5 to 3.5 internal diameter (ID) endotracheal tubes (ETTs). This study implemented multiple system components that were previously developed [47, 48, 59, 61], and now assembled into the iDP-ADS. The EM Timer consisted of a digital pressure regulator, micro-solenoid valves and a timer control unit (Fig. 1b). The outlet flow rate of compressed air (*in vitro* testing) or pure oxygen (*in vivo* aerosol delivery) was set to approximately 3 L/min and the timer was set to provide either 10 mL (*in vitro* testing) or approximately 7 mL/kg (*in vivo* aerosol delivery) of gas actuation volume (GAV). The air-jet DPI was characterized by three inlet flow passages arranged in a triangular pattern (0.5 mm inlet diameters), a 0.68 mL aerosolization chamber in an approximate capsule shape, and a single 0.89 mm stainless steel capillary outlet (Fig. 1c). The capillary outlet tube had an outlet length of 6.7 cm and was connected to a diffusional flow pathway, as with the gradual expansion illustrated in Fig. 1c, which then led to an ETT interface connection.

The PMC unit (Fig. 1d) served the multiple purposes of applying a resistance during animal exhalation to help maintain positive end expiratory pressure (PEEP), monitoring pressure near the ETT inlet, and providing pop-off valve pressure relief if ETT pressure exceeded approximately 25 ± 2 cm H_2_O. As illustrated in Fig. 1, the PMC unit was connected in the diffusional flow pathway very near the ETT to minimize additional rebreathing dead space (< 0.5 mL). Connections from the PMC central region led to an analog pressure gauge (AG Industries, St. Louis, MO) and a pop-off valve (Ambu 22 mm PEEP valve, Ballerup, Denmark). The top of the PMC was a flow resistor cap with a 0.5 mm diameter central aperture, which served as an exhalation port. For the *in vivo* studies, the operator covered this exhalation port with their thumb during device actuation and a brief breath-hold period (~ 1 s), and then released the port to allow exhalation. By observing the pressure gauge, the exhalation port was covered near the end of exhalation to maintain ~ 5 cm H_2_O of PEEP in the lungs prior to the next device actuation.

**Fig. 2:**
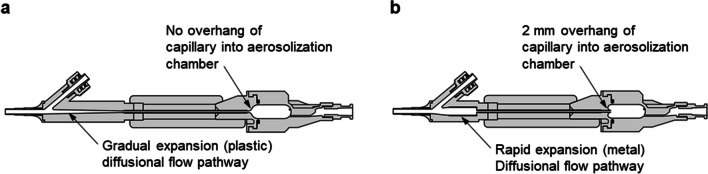
Aerosolization engines and diffusional flow pathways for (**a**) Design 1 (D1) and (**b**) D2 air-jet DPIs.

In this study, two versions of the air-jet DPI were considered, which are referred to as Designs 1 and 2. In Design 1 (D1; Fig. 2a), the outlet of the aerosolization chamber was flush with the chamber walls. Preliminary testing of this device indicated that best aerosolization performance occurred with maximum 10 mg powder loadings and the aerosol emptied very quickly (within three actuations and mostly on the first actuation). Based on previous device development work [48], a gradual expansion (GE) flow diffuser was used to dissipate the high velocity aerosol flow prior to entry into the ETT.

In order to potentially increase loaded dose and improve aerosol quality, a new Design 2 (D2) air-jet DPI was also considered (Fig. 2b). With the D2 device, the outlet flow passage (capillary) protruded into the aerosolization chamber approximately 2 mm and was smoothly connected to the remainder of the aerosolization chamber using continuously curving walls. The intent of this design was to slow the release of the aerosolized powder and reduce the size of the particles that could exit the chamber while not increasing velocity in the outlet flow passage. In D2 the GE flow diffuser of D1 was replaced with a rapid expansion (RE) geometry (Fig. 2b). This change was made to potentially reduce the occurrence of aggregate buildup and re-entrainment that was expected to occur in the GE of D1.

### Development of Rabbit Lung Chamber (LC) Model

**Fig. 3:**
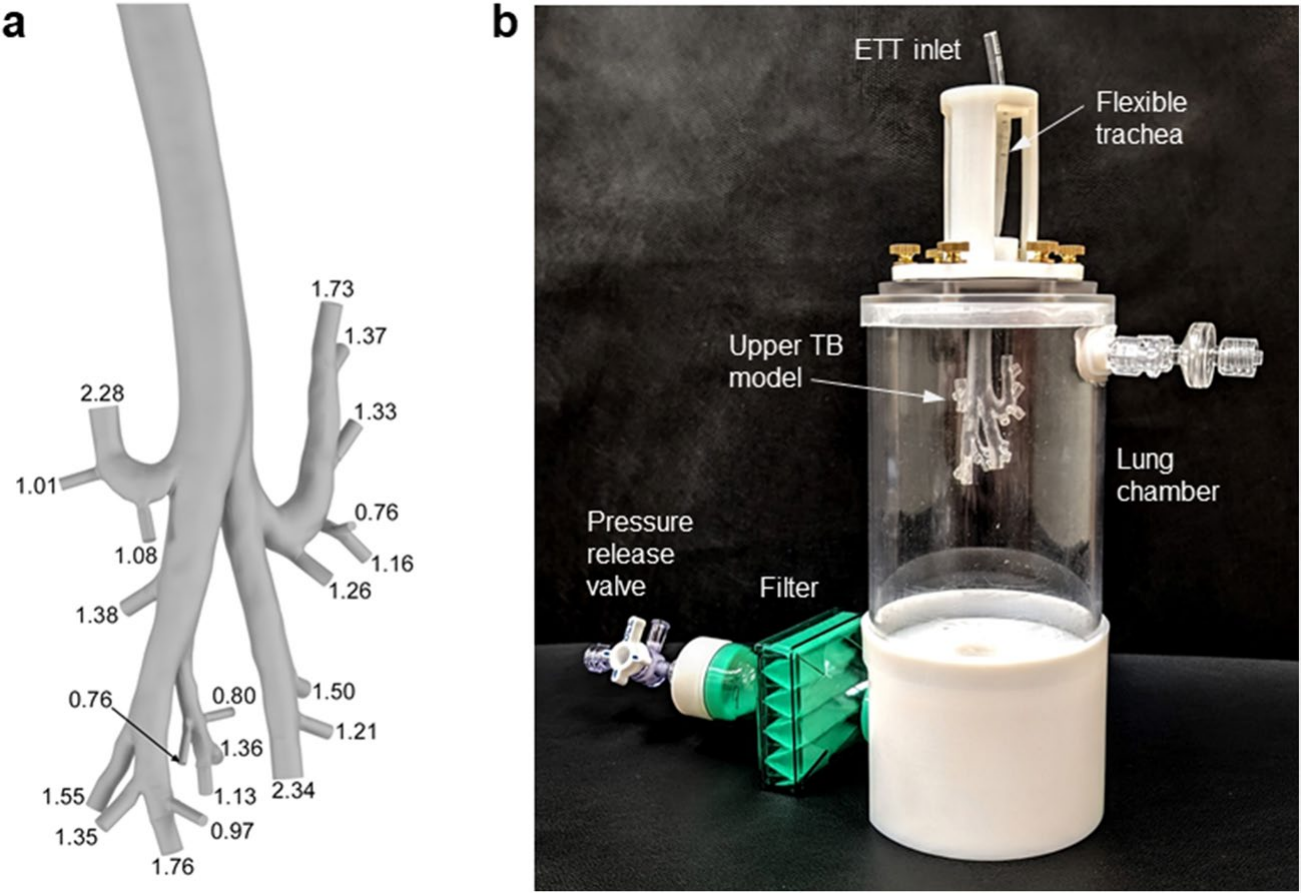
*In vitro* models used for realistic aerosol delivery testing including the (**a**) upper tracheobronchial (TB) airway geometry with outlet diameters (in millimeters) labeled, and (**b**) complete Rabbit Lung Chamber (LC) Model.

To test lung delivery of the dry powder AST product when administered through an ETT to an infant-size test animal, an *in vitro* model of an approximate rabbit upper tracheobronchial (TB_upper_) region surrounded by a cylindrical lung chamber (TB_upper_ + LC) was generated, as shown in Fig. 3. This model was used to predict aerosol deposition in the upper TB region (starting with the trachea and extending through approximately 4–6 bronchial generations, or G4-G6), delivery beyond the upper TB region, and to provide approximate airway resistance and compliance during aerosol testing. To form the upper TB animal airway, a cast or scan of rabbit airways was not directly available. As an alternative, we selected a pre-existing cast of a similar size small mammal (ferret airway [69]; provided by Dr. Robert Phalen) that had airway dimensions very similar to young mature New Zealand White Rabbits. Specifically, Hack and Gearhart [70] reported the mean diameters for the first three generations of mature rabbit airways beginning with the trachea to be 0.53, 0.42 and 0.26 cm, respectively. We selected the young adult Fe#4 model of Oldham *et al*. [69] with corresponding airway diameters of 0.52, 0.38, and 0.29 cm, respectively. Airway lengths were also similar, with the exception of the trachea, which was conservatively longer (i.e., producing higher deposition) in the ferret model (9.4 cm ferret vs. 5.6 cm rabbit). An original young adult ferret TB silicone cast from the study of Oldham *et al*. [69] was translated to a digital object using a Bruker SkyScan 1173 microCT scanner (Bruker Corporation, Billerica, MA) and Slicer 4.10.2 software (www.slicer.org) [71]. Based on available scan resolution, the final upper TB airway model contained pathways with between 3 to 5 bifurcations, or 4 to 6 generations, where the trachea was counted as generation G1. Final outlet diameters ranged between 2.34 mm and 0.76 mm and are illustrated in Fig. 3a.

A majority of the trachea (~ 5 cm) was constructed in cast silicone to provide realistic insertion conditions for the ETT interface. The remainder of the upper TB airway was constructed as a hollow body and 3D printed by Quickparts (Seattle, Washington) using SLA technology with Accura ClearVue resin. Considering that a majority of airway resistance resides in the upper TB region, this upper airway section was used to approximate the expected airway resistance during ETT aerosol delivery.

The complete Rabbit LC Model is illustrated in Fig. 3b with the LC surrounding the TB geometry. The main body of the LC was constructed using a clear acrylic cylinder with a volume of ~ 400 mL to approximate the expected *in vivo* post-washout rabbit lung compliance of approximately 0.4–0.6 mL/cm H_2_O. Volume of the clear cylinder, outlet pathway and filter housing produced a total chamber compressible volume of ~ 500 mL, and resulted in a measured compliance of 0.45 mL / cm H_2_O. The outlet pulmonary filter (Pulmoguard II™, Queset Medical, North Easton, MA) was used to capture aerosol that did not deposit in the upstream sections. During aerosol testing, a final three-way valve was used to release the chamber pressure after each delivery cycle while capturing the aerosol on the filter, and for connection to a downstream vacuum pump for a final purge of the chamber after the last actuation, if needed. A pressure measurement port was also included on the chamber side wall for measurement of the LC pressure conditions.

### Air-Jet DPI Recommended Settings and Pressure Testing in the Rabbit LC Model

During aerosol delivery, the rabbit surfactant washout *in vivo* model was ventilated by the air-jet DPI (with aerosol contained in each breath). As a result, delivered gas conditions were critical to maintain oxygenation of the animal and to avoid additional lung damage, either through pressure trauma, volume trauma, or lung collapse (atelectotrauma). As an initial guide consistent with the animal model ventilator settings, the air-jet DPI delivered approximately 7 mL/kg GAV with each inhalation. A challenge with using a fixed tidal volume (per kg) in all cases was that as lung compliance decreases with surfactant washout, the administered pressure required to achieve 7 mL/kg increases, often exceeding safe levels.

For a set amount of delivered gas volume (represented as GAV or more commonly as tidal volume, V_t_; mL) and inlet flow rate (Q_in_; L/s) as provided by the iDP-ADS, the maximum pressure sensed by the device (P_D_) at the PMC unit is the sum of the airway elastic pressure contribution (∆P_E_), resistive pressure contribution (∆P_R_), and PEEP present at the end of the previous breath, where P_D_ can be calculated as

1 $${P}_{D}={\Delta P}_{E}+{\Delta P}_{R}+PEEP=\frac{1}{C}{\cdot V}_{t}+R\cdot {Q}_{in}+PEEP.$$

In Eq. (1), C is the lung average compliance (mL / cm H_2_O) and R is the interface and airway total resistance (cm H_2_O / L/s). The resistive pressure term is largely dissipated (by viscous shear stress) in the interface and conducting airways and represents the pressure loss required to deliver the gas through these conducting pathways to the alveolar region at the specified flow rate. Pressure within the lungs becomes a transient volume filling problem that reaches a steady state equilibrium within 3–4 time constants (τ), where τ = R C. For the monopodial small-mammal airway structure and ETT delivery, measured R was relatively small (i.e., 4 cm H_2_O / L/s). As a result, the administration time of ~ 0.2 s represents > 10 time-constants, indicating little delay between device and lung pressures. Similarly, there is negligible difference between device and LC pressure conditions, which was also verified experimentally (< 0.3 cm H_2_O). For the gas delivery conditions (i.e., constant Q_in_ and V_t_), the maximum airway pressure (P_AW_) in the alveolar region, approximated with the LC pressure, can therefore be represented as:

2 $${P}_{AW}\approx {P}_{D}\approx {\Delta P}_{E}+PEEP=\frac{1}{C}{\cdot V}_{t}+PEEP.$$

During aerosol delivery, pressure targets were set as an elastic pressure rise (∆P_E_) of  ≤ 20 cm H_2_O and a PEEP value of  ~ 5 cm H_2_O. Maximum and minimum device and LC pressures should then be in a range of approximately 25 ± 2 to 5 ± 2 cm H_2_O. Given the very close similarity in LC and device pressures, only LC pressures were recorded and reported.

To enable rapid setting of the gas delivery conditions and achieve the targeted pressure range, for variable test animal weights and lung compliances, Table I was generated. The pressure regulator was preset and calibrated to provide a flow rate of 3 L/min (0.05 L/s). For a selected rabbit weight, Table I provided the baseline timer settings for entry into the EM Timer device to provide approximately 7 mL/kg tidal volumes. Grey shading was used in Table I to indicate the change in pressure at different airway compliances based on the elastic effect alone (∆P_E_). The actual ∆P_E_ was also available in Table I. For ∆P_E_ values  > 20 cm H_2_O (light and dark grey), it was recommended that the timer setting be reduced to the first row of that compliance column with a value that would produce a ∆P_E_ ≤ 20 cm H_2_O (no shading). For example, for a 1.6 kg rabbit with a lung compliance of 0.45 mL / cm H_2_O, the baseline tidal volume of 7 mL/kg would generate a ∆P_E_ of 25.0 cm H_2_O. At this compliance, it is recommended that the aerosol delivery time from the DPI be reduced to 0.18 s generating a ∆P_E_ of 20 cm H_2_O and an acceptable tidal volume of 5.7 mL/kg.

**Table I:**
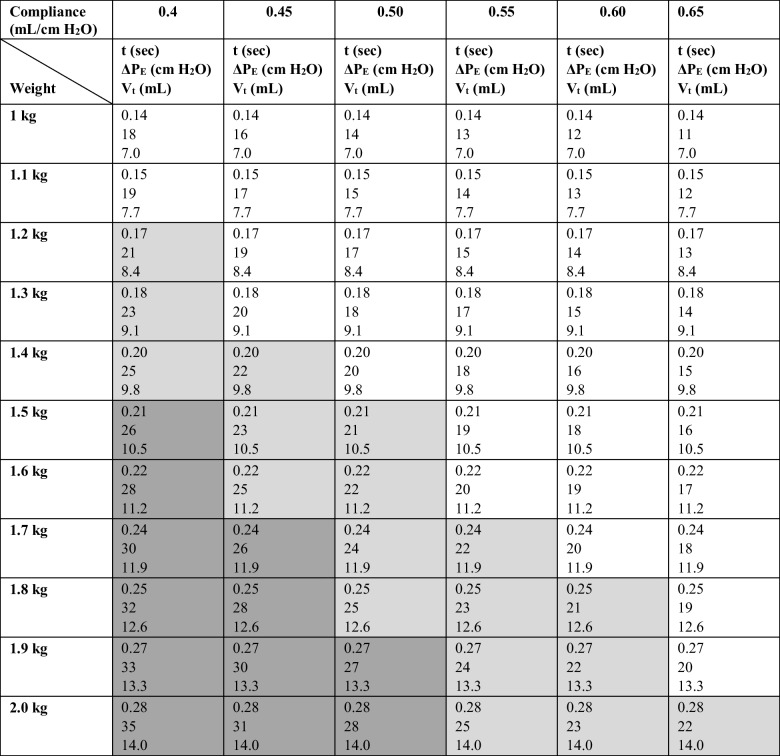
EM Timer Settings (t), Predicted Change in Elastic Pressure (ΔP_E_) and Administered Tidal Volume Across a Range of Lung Compliances for an Air-Jet DPI Delivered Flow Rate of 3 L/min and Targeted Tidal Volume of 7 mL/kg

Light Grey Cells: Predicted to create a ΔP_E_ of > 20 cm H_2_O; total administered pressure may exceed 25 cm H_2_O; operator should consider reducing actuation time by moving up to the first clear cell in the same column

Dark Grey Cells: Predicted to create a ΔP_E_ of > 25 cm H_2_O; total administered pressure may exceed 30 cm H_2_O; operator should consider reducing actuation time by moving up to the first clear cell in the same column

To test administered lung pressures, the delivery device was connected to the Rabbit LC Model, with a measured lung compliance of 0.45 mL/cm H_2_O, and operated in a similar manner with the planned *in vivo* experiments. The LC pressure relief valve was maintained in the closed position and the PMC exhalation port was used to release each delivered breath, after the brief breath hold. Pressure profiles were recorded at the lung chamber pressure port (LC pressure). The dry powder formulation was not delivered during LC pressure testing experiments. The respiration rate for controlling actuation of the EM Timer depended on the GAV time, an approximate 1-s breath hold, plus the exhalation time required for the chamber to reach the desired PEEP pressure of 5 ± 2 cm H_2_O. Pressure testing conditions were:

- Scenario 1: 1.3 kg rabbit; 0.45 mL / cm H_2_O; 0.18 s timer setting (7 mL/kg V_t_)
- Scenario 2: 1.6 kg rabbit; 0.45 mL / cm H_2_O; 0.22 s timer setting (7 mL/kg V_t_)
- Scenario 3: 1.6 kg rabbit; 0.45 mL / cm H_2_O; 0.18 s timer setting (5.7 mL/kg V_t_)

Based on estimated conditions, Scenario 1 should satisfy the desired ∆P_E_ and LC pressure requirements; Scenario 2 represents selection of 7 mL/kg for a 1.6 kg rabbit without adjusting for lung compliance; and Scenario 3 provides the correct settings for a 1.6 kg rabbit considering both tidal volume and lung compliance that satisfies the pressure requirements.

### Aerosol Size Characterization using Cascade Impaction

The air-jet DPI aerosol characterization experiments utilized a Next Generation Impactor (NGI; MSP, TSI Incorporated, Shoreview, MN) for aerosol particle size analysis. To assess the aerosol size distribution, the air-jet DPI (with the ETT attached to the outlet to assess size of the lung delivered dose) was connected to the pre-separator inlet of the NGI using a custom adapter, as previously described by Howe *et al*. [46]. The adapter positioned the ETT exit approximately 1 cm away from the center of the pre-separator inlet with open space allowing for co-flow room air to enter the NGI, which was operated at a constant flow rate of 45 L/min using a downstream vacuum pump; while the device was operated with the intended 3 LPM flow rate. The NGI was positioned 90° off horizontal using an angle guide to allow the device to remain level during use (as intended for a horizontal infant or test animal) and maintained an inline flow path from the device outlet to the NGI inlet. Each stage of the NGI was coated with MOLYKOTE® 316 silicone spray (Dow Corning, Midland, MI) to minimize particle bounce and re-entrainment. The NGI flow rate of 45 L/min was chosen to ensure collection of the aerosol, minimize any effects of settling and provide appropriate stage cutoff diameters for evaluating small aerosol sizes. Before each set of experimental runs, the flow rate was confirmed using a flow sensor (Sensirion SFM3000, Sensirion AG, Stafa, Switzerland) connected to the NGI inlet.

The air-jet DPI was loaded with 10, 20 or 30 mg of the Nano SD SLS-EEG formulation and actuated into the NGI via the EM Timer air source using multiple (three or more) 10 mL GAVs. The flow rate and actuation time were 3 L/min and 0.2 s, respectively. For each device and loaded formulation mass, the device was actuated until exiting aerosol was no longer visible, followed by one additional actuation. Three replicate runs for each experiment were performed and quantitative analysis of DPPC deposition using the HPLC–UV assay was used to assess the aerosol performance characteristics of the SLS-EEG powder formulation. Analysis metrics included emitted dose (ED), mass median aerodynamic diameter (MMAD), fine particle fraction (FPF) and geometric standard deviation (GSD). Emitted dose was calculated as the mass of DPPC in the loaded dose minus the mass of DPPC remaining in the device divided by the initial loaded mass of DPPC. Aerosol size calculations were based on the mass of DPPC recovered in the NGI (including pre-separator). Based on an airflow rate of 45 L/min, the NGI stage cut-off diameters were determined using the formula specified in USP 35 (Chapter 601, Apparatus 5). The mass median aerodynamic diameter (MMAD) and fine particle fractions (FPFs < 5 µm or < 1 µm) were calculated using Copley Inhaler Data Analysis Software (CITDAS).

### *In Vitro* Testing of Lung Delivery Efficiency with Rabbit LC Model

The Rabbit LC Model *in vitro* system was used to conduct realistic aerosol delivery testing and training for use of the air-jet DPI. To evaluate regional aerosol deposition in the LC Model, the inner walls of the airway model were coated with MOLYKOTE® 316 silicone spray (Dow Corning, Midland, MI) to minimize particle bounce and simulate an airway fluid coating. The air-jet DPI was loaded with 10 or 20 mg of the SLS-EEG formulation and connected to the ETT, which was inserted into the flexible silicone trachea. The LC Model was positioned horizontally on a flat surface, similar to the orientation of the rabbits in the *in vivo* experiments. The air-jet DPI was actuated for multiple cycles (typically three times for D1 and five times for D2) with the LC pressure monitored and released after each actuation. After the air-jet DPI ETT connection was removed, the final pressure chamber evacuation was performed to capture any remaining suspended aerosol on the filter using a downstream vacuum pump. Deposited powder formulation was recovered from the device, ETT, upper TB model, chamber and filter using appropriate wash volumes of methanol and those solutions were analyzed by HPLC–UV to determine the DPPC content. The chamber and filter deposition fractions were grouped together as a lower lung deposition fraction. The upper TB, chamber and filter deposition fractions were grouped together as a total lung deposition fraction. All deposition fractions were expressed as a percentage of device nominal loaded dose based on the HPLC quantification of DPPC and were calculated from loaded powder mass and previously determined DPPC content.

Previous studies of dry powder surfactant aerosol delivery have noted significant reductions in lung delivery efficiency of aerosol when realistic humidity conditions are included [72]. With EEG aerosols, it is important to establish that overly rapid aerosol growth does not occur in the upper TB region thereby significantly reducing the fraction delivered to the deep lung. Based on the small number of actuations required to deliver the aerosol with the air-jet DPI [3–5 actuations expected], we anticipate that the GAV can be either dry or at room humidity and temperature conditions with minimal impact on the lungs. To capture the impact of lung humidity conditions near 100% RH, experiments (using 20 mg device loaded mass) were conducted in which the walls of the LC Model were pre-wetted. Briefly, realistic humidity conditions (37 ± 2°C and 98 ± 2% RH) were created in an environmental chamber (ESPEC Corp., Grand Rapids, Michigan) and the Rabbit LC Model equilibrated in the chamber. Heated and humidified air was passed through the ETT to upper TB and LC regions until the walls of the system were saturated, with visibly wet surfaces. Large condensed water droplets were visible in the ETT, upper TB model and LC walls. The SLS-EEG formulation delivery was repeated as previously described into the humified Rabbit LC Model.

### Statistical Analysis

Statistical analysis for comparing aerosol delivery performance and estimated lung delivery efficiencies were performed using JMP Pro 17 (SAS Institute Inc., Cary, NC). The NGI particle size distribution and regional deposition data between D1 and D2 device were compared using Student’s t-Test while comparison of multiple cases (effect of different loaded mass on NGI deposition fractions) utilized one-way ANOVA followed by post hoc Tukey. Statistical tests used a significance limit of *p* = 0.05.

## Results

### Formulation Characterization: DPPC and B-YL Content Uniformity

Formulation content uniformity in terms of DPPC and B-YL content were assessed using validated HPLC–UV methods. Over the concentration ranges (50–400 μg/mL for DPPC and 7–25 μg/mL for B-YL), for both DPPC and B-YL, the calibration curves were linear with correlation coefficients (R^2^) ≥ 0.998. The accuracy (%relative error) in the linear concentration range was ≤ 9.6% for DPPC and ≤ 4.6% for B-YL. The precision (%CV) in the linear concentration range was ≤ 7.0% for DPPC and ≤ 7.7% for B-YL. The limit of quantitation (LOQ) for DPPC and B-YL was 6.8 µg/mL and 4.0 µg/mL, respectively.

Over the course of the study, multiple batches of the Nano SD SLS-EEG formulation were produced. The mean (SD) DPPC content of seven batches of spray-dried synthetic lung surfactant powder formulation was 50.5 (1.8) %w/w, which was 96.6% of the nominal content (52.5%). The coefficient of variation for DPPC content was 3.5%, indicating good reproducibility in DPPC content across batches. The mean (SD) B-YL content was 2.8 (0.0) %w/w, which was 55.9% of the nominal content (5%). The lower measured content of B-YL in the formulation may have been due to peptide degradation during the mesh-nebulizer-based spray drying process. Analysis of the feed solution indicated that the nominal amount of B-YL was present prior to and after completion of the spray drying. Interestingly, an identical formulation spray-dried using the nozzle based Buchi S300 spray dryer did not have any change in the B-YL content (5.1 (0.1) %w/w) following spray drying, indicating the loss of B-YL was likely specific to the Buchi B-90 HP spray dryer.

### Formulation Characterization: Laser Diffraction Particle Sizing

Evaluation of the particle size of the SLS-EEG spray-dried formulation is shown Table II with the volumetric particle size determined at dispersion pressures of 0.5 and 4.0 bar for seven different batches. The mean primary particle size (Dv50) of the SLS-EEG formulation batches measured at 4 bar dispersion pressure was 0.82 ± 0.02 µm with 100% of particles < 5 µm. Significantly, there is a large submicrometer fraction with 57.8% of particles being less than 1 µm. The coefficient of variation for the Dv50 was 2% reflecting the consistent size of the spray dried SLS-EEG formulation for the seven batches considered and reproducibility of the powder preparation process. The mean Dv50 of the batches measured at 0.5 bar dispersion pressure was 1.32 ± 0.22 µm, suggesting good dispersibility of the powders. The spray drying process produces micrometer-sized particles suitable for deep lung delivery, which were nearly fully dispersed to primary particles using the high dispersion pressure. Some small degree of aggregation was observed when the lower dispersion pressure was employed and this dispersion was associated with greater variability; however, the mean (Dv50) powder formulation size of 1.32 µm remained suitable for the EEG approach and deep lung delivery.

**Table II:** Mean ± SD Particle Size Parameters of the Nano SD Micrometer-Sized SLS-EEG Formulation at 4.0 and 0.5 Bar Dispersion Pressures (data are mean ± standard deviation of 7 different batches). Values in Parenthesis Represent the Coefficient of Variation

Dispersion pressure	Particle size		
	Dv10 (µm)	Dv50 (µm)	Dv90 (µm)
4.0 bar	0.49 ± 0.01 (2%)	0.82 ± 0.02 (2%)	1.50 ± 0.08 (5%)
4.0 bar	0.60 ± 0.03 (5%)	1.32 ± 0.22 (16%)	3.57 ± 1.18 (33%)

### Formulation Characterization: Scanning Electron Microscopy

**Fig. 4:**
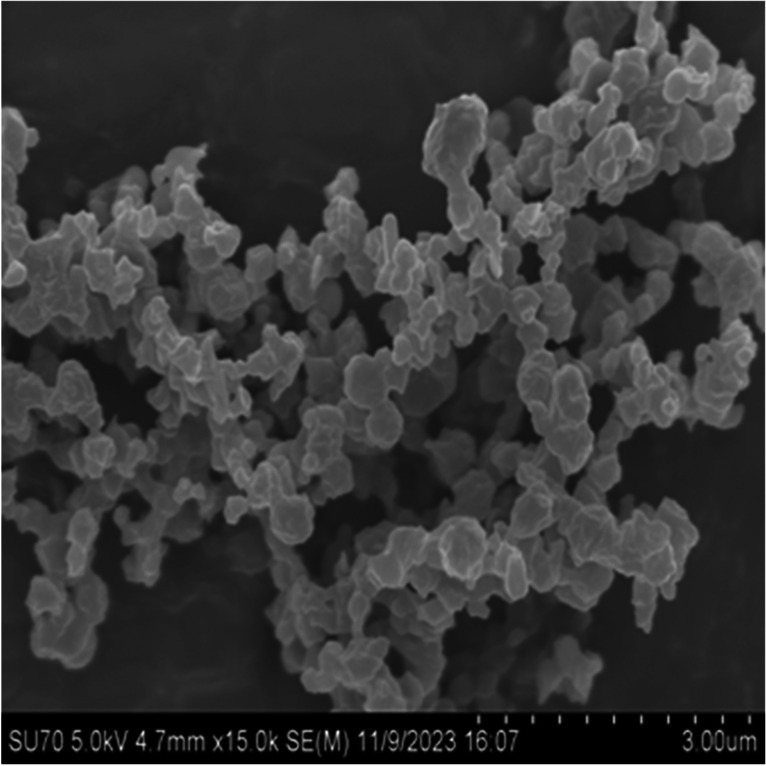
Scanning electron micrograph of spray-dried SLS-EEG dry powder particles at 15k magnification.

Figure 4 shows the scanning electron micrographs of the spray-dried SLS-EEG powder formulation and indicates good agreement with the laser diffraction measurements. In general, the powder particles were spherical with wrinkled surfaces, which is clearly visible at high magnification (15K).

### Formulation Characterization: XRPD and Moisture Sorption

**Fig. 5:**
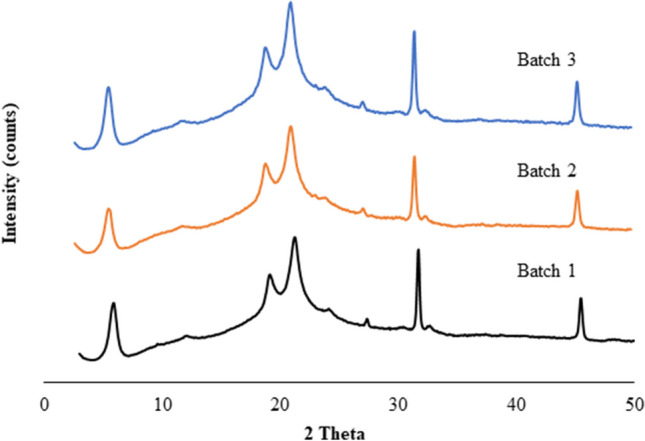
X-ray powder diffractograms of the SLS-EEG powder.

The solid-state properties of the SLS-EEG powder formulations were characterized using XRPD and water vapor moisture sorption. Figure 5 shows the X-ray powder diffractograms of three different batches of the SLS-EEG formulation. X-ray powder diffraction showed evidence of a partially crystalline structure with peaks corresponding to mannitol and l-leucine. There were no obvious differences in the peak diffractograms indicating reproducible particle production between the batches.

**Fig. 6:**
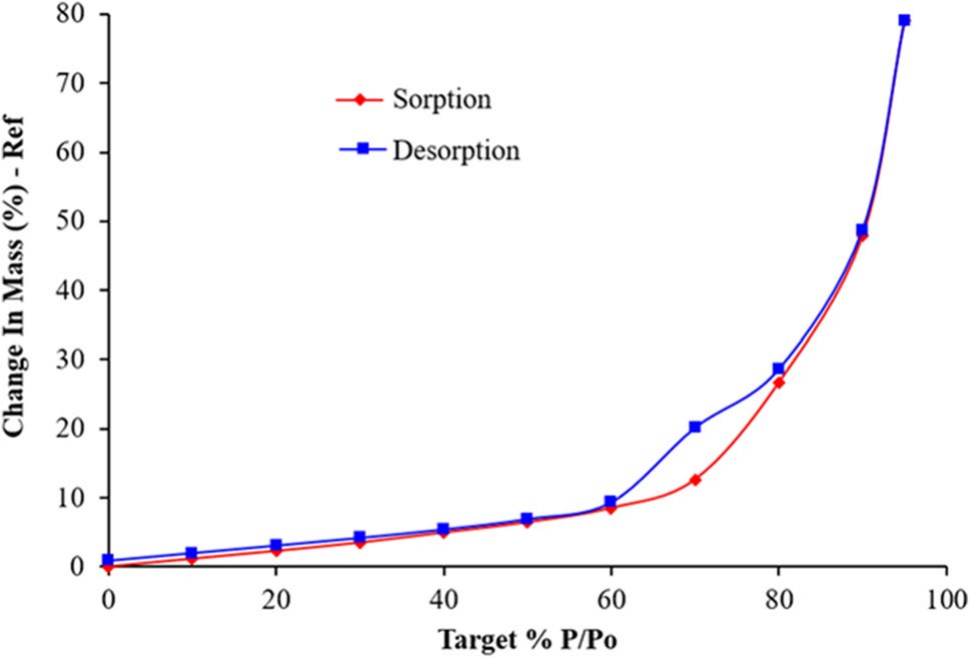
Dynamic vapor sorption behavior of the SLS-EEG powder.

The water vapor sorption behavior of the SLS-EEG powder measured with DVS is shown in Fig. 6. Following drying of the powder formulation during an equilibration period at 0% RH, water uptake by the powder under ambient-like conditions (10–60% RH; red line) was less than 9%, indicating relatively low uptake for an unprotected spray dried powder (Fig. 6). Above 60% RH, the moisture uptake increased significantly, indicating the hygroscopic nature of the formulation once the l-leucine barrier layer is overcome at elevated RH. This biphasic behavior is well suited for the EEG dry powder aerosol application. Specifically, the water vapor sorption behavior indicates low moisture uptake during storage under ambient conditions and elevated uptake at higher humidity similar to the airway environment when hygroscopic growth in the humid lung airways is required for EEG particle size growth to significantly enhance particle deposition and retention.

### Air-Jet DPI Pressure Testing in the Rabbit LC Model

**Fig. 7:**
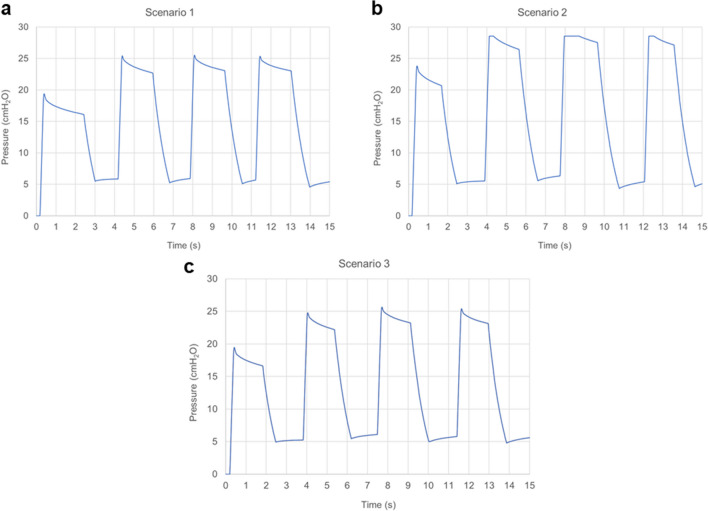
Pressure waveforms measured within the Rabbit LC Model for gas delivery scenarios (and maximum pressures): (**a**) 1 (25.9 cm H_2_O), (**b**) 2 (28.6 cm H_2_O), and (c) 3 (25.7 cm H_2_O).

Pressure testing in the Rabbit LC Model was conducted based on three aerosol delivery scenarios described in the Methods with the use of the EM Timer settings provided in Table I. For Scenario 1 delivery (1.3 kg rabbit; 0.45 mL / cm H_2_O; 0.18 timer setting), Table I predicts that the ΔP_E_ will meet the ≤ 20 cm H_2_O requirement at a tidal volume of 7 mL/kg. The measured LC pressure (Fig. 7a) illustrates the first administered breath (starting from zero-gauge pressure in the lung chamber) followed by three additional breath cycles. In each case, ΔP_E_ falls within the ≤ 20 cm H_2_O range, together with 5 cm H_2_O PEEP, providing a total LC pressure ≤ 25 ± 2 cm H_2_O, as targeted. The operator was also able to capture the 5 cm H_2_O PEEP with each cycle and apply the approximately 1-s recommended breath hold. For Scenario 2 (1.6 kg rabbit; 0.45 mL / cm H_2_O; 0.22 s timer setting), as indicated in Table I, the measured ΔP_E_ was approximately 25 cm H_2_O, and when combined with 5 cm H_2_O PEEP, produced a total LC pressure that was out of the desired range (i.e., > 25 ± 2 cm H_2_O Fig. 7b). In some cases, the administering clinician may wish to prioritize maintaining an administered tidal volume of 7 mL/kg and accept the higher administered pressure for a limited number of breaths, for example to prioritize lung recruitment. In cases where the clinician wishes to prioritize maintaining the lung pressure ≤ 25 ± 2 cm H_2_O, Scenario 3 adjustments should be applied. In this case, the clinician moves up the 0.45 mL / cm H_2_O compliance column in Table I to the first clear cell, which administers a ΔP_E_ of 20 cm H_2_O based on reducing the EM Timer setting to 0.18 s. The resulting total tidal volume is 9.1 mL, which for the 1.6 kg subject results in a weight-based tidal volume of 5.7 mL/kg. As indicted in Fig. 7c, when applied to the Rabbit LC Model the Scenario 3 measured LC pressure was then ≤ 25 ± 2 cm H_2_O, meeting the specified lung pressure recommendation.

### Aerosol Size Characterization: Comparison of D1 and D2 Designs

Table III provides the aerosol size distribution of the Nano SD SLS-EEG formulation determined by using the Next Generation Impactor (NGI) and Copley Inhaler Testing Data Analysis Software (CITDAS) after delivering 10 mg loaded mass from the D1 and D2 devices. The powder formulation device retention (as measured by DPPC assay) for the D2 device (18.8%) was significantly higher than the D1 device (5.6%) (p < 0.05). However, the pre-separator deposition, the fraction of the aerosol greater than ~ 13 µm, for the D2 device was about 20% (absolute difference) lower than the D1 device (38.4% vs 60.8%) (*p* < 0.05), suggesting more efficient aerosolization using the D2 device. This agreed well with the measured MMAD, which revealed that the D2 device produced a significantly smaller MMAD than the D1 device (1.7 µm vs 2.3 µm). Correspondingly, the FPF_<5 µm_ for the D2 device was also higher than the D1 device (37% vs 21%) (*p* < 0.05). The observed differences between the micrometer-size primary particles measured by Sympatec and the measured MMADs reflect incomplete deaggregation of the primary particles at the lower dispersion pressures used in the air-jet DPI. The implemented inhaler design modifications improved the aerosol size characteristics of the emitted aerosol with only a small impact on the overall emitted dose. Given the smaller aerosol size (MMAD) and acceptable emitted dose (80%) properties observed with NGI testing, the D2 device was selected for the subsequent dose planning study, described below.

**Table III:** Aerosol Size Distribution of Nano SD SLS-EEG Formulation Determined by Using Next Generation Impactor (NGI) and Copley Inhaler Testing Data Analysis Software (CITDAS) After Delivering 10 mg Loaded mass from the D1 and D2 Devices (data are means ± standard deviations, *n* = 3; MMAD: mass median aerodynamic diameter, FPF: fine particle fraction)

Sections and terms	D1	D2
Device retention (%)	5.6 ± 0.5	18.8 ± 1.9*
Pre-separator fraction (%)	60.8 ± 1.3	38.4 ± 1.3*
MMAD (µm)	2.3 ± 0.2	1.7 ± 0.3*
FPF < 1 µm (%)	8.1 ± 2.3	12.0 ± 4.3
FPF < 5 µm (%)	20.9 ± 2.5	37.4 ± 1.5*
Emitted Dose (%)	94.4 ± 0.5	81.2 ± 1.9*
Total recovery (%)	96.4 ± 1.4	92.7 ± 0.6

^*^Significant difference in comparison to D1; Student’s t-test, *p* < 0.05

### Dose Planning: Impact of Higher Loaded Doses

Two additional SLS-EEG formulation doses (20 and 30 mg) were used to evaluate the impact of loaded dose on aerosol performance from the D2 device prior to *in vivo* testing (Table IV). Loaded dose mass had a significant effect on device retention and NGI sizing. At 30 mg loaded mass, the emitted dose (87.2%) was higher than with 10 mg (ED = 81.2%) and 20 mg (ED = 78.4%) loaded masses (Table IV). However, no differences were found in the emitted doses of between 10 and 20 mg loaded mass (p > 0.05). With increase in loaded mass, the pre-separator fraction also increased (p < 0.05), which contributed to the decrease in fine particle fractions less than 5µm and 1µm (Table IV). Considering a need to maintain an ED of ~ 80% or higher, an MMAD preferably < 2 µm and maximum FPFs, loaded dose masses of 10 and 20 mg with the D2 device were selected for additional testing in the Rabbit LC Model.

**Table IV:** Deposited Mass Fraction of Nano SD SLS-EEG Formulation after Delivering Different Loaded Masses to Next Generation Impactor (NGI) Using the D2 Device (data are means ± standard deviations, n = 3)

Sections and terms	Loaded mass		
	10 mg	20 mg	30 mg
Device retention (%)*	18.8 ± 1.9	21.6 ± 1.7	12.8 ± 1.8^
Pre-separator fraction (%)*	38.4 ± 1.3	44.4 ± 1.5^	54.4 ± 0.7^
MMAD (µm)*	1.7 ± 0.3	1.9 ± 0.1	2.9 ± 0.5^
FPF < 1 µm (%)*	12.0 ± 4.3	9.3 ± 0.5	6.4 ± 0.7^
FPF < 5 µm (%)*	37.4 ± 1.5	27.1 ± 1.5^	19.3 ± 0.4^
Emitted Dose (%)*	81.2 ± 1.9	78.4 ± 1.7	87.2 ± 1.8^
Total recovery (%)	92.7 ± 0.6	95.5 ± 1.7	94.3 ± 2.1

^*^p < 0.05 significant effect of loaded mass on deposition fraction (one-way ANOVA)

^*p* < 0.05 significant difference compared to 10 mg loaded mass (post-hoc Tukey)

### *In Vitro* Rabbit LC Model: Performance of the D2 Design

**Fig. 8:**
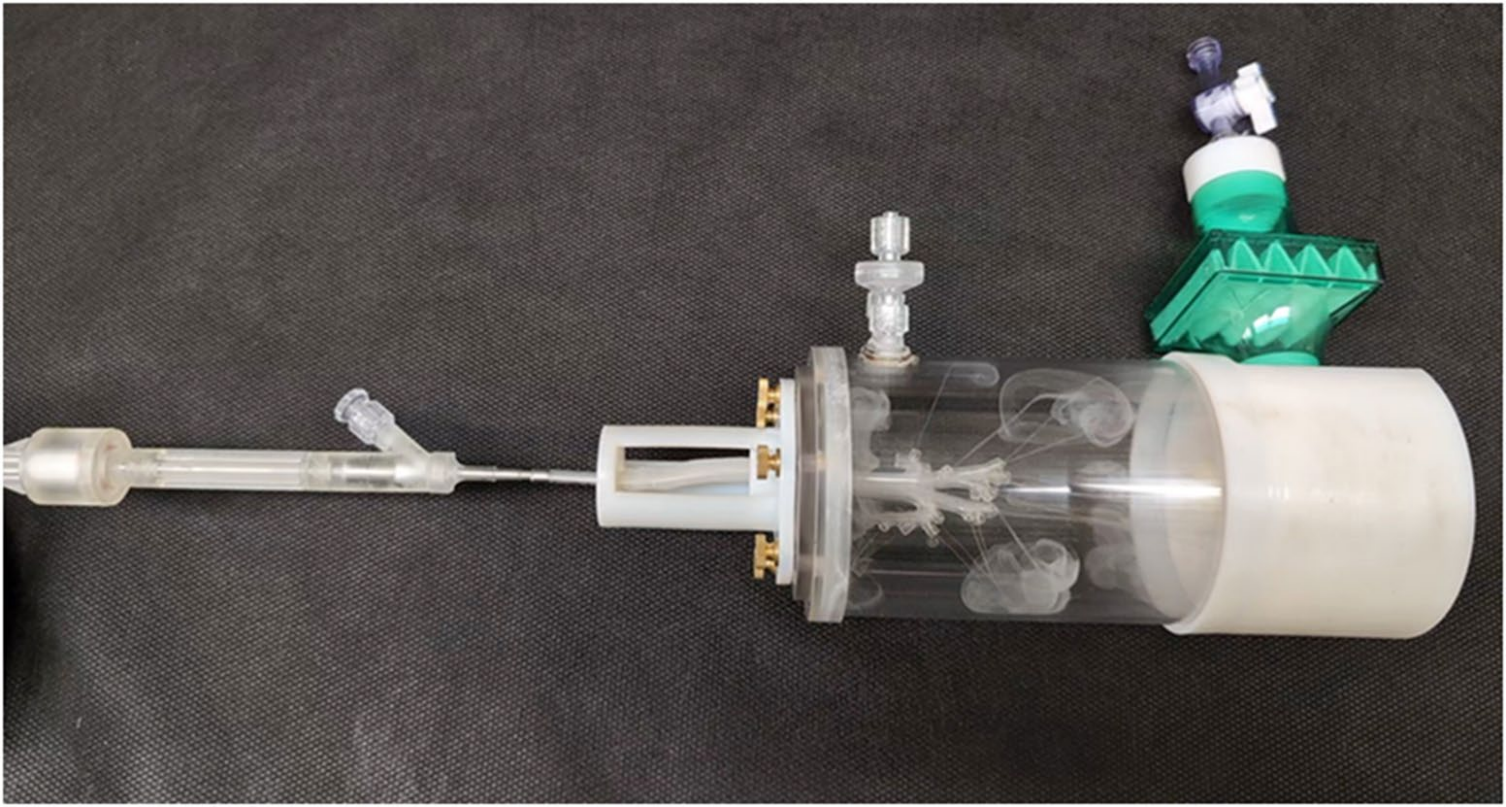
Setup for *in vitro* testing of lung delivery efficiency with Rabbit LC Model showing the powder aerosolization (milliseconds after the first actuation; D2 device) and orientation of the LC model similar to the rabbits in the *in vivo* experiments (cf. Part II).

The *in vitro* regional deposition fractions and estimated rabbit lung delivery efficiencies of SLS-EEG formulation were assessed using the *in vitro* Rabbit LC Model when delivered from the D2 device, as illustrated in Fig. 8, and the results are summarized in Table V. Considering the D2 device loaded with 10 mg of SLS-EEG formulation, the emitted dose was 80.4%, which was similar to the value that had been observed previously in the NGI studies. Aerosol deposition in the ETT and TB region was low, with only 3% of the aerosol being deposited in the 3 mm ETT (Table V). High fractions of the loaded dose were delivered to the simulated rabbit lungs, in terms of both the lower lung dose (filter + lung chamber) and total lung dose (TB + filter + lung chamber) delivery with values of 57.3% and 72.7%, respectively, using the D2 device and 10 mg loaded dose.

**Table V:** Regional Deposition Fractions (%) and Lung Delivery Efficiencies of Different Loaded Masses of Nano SD SLS-EEG Formulations Delivered from the D2 Device to the *in vitro* Rabbit LC Model (data are means ± standard deviations, *n* = 3)

Sections and terms	Deposited mass (%)		
	10 mg	20 mg	20 mg (humidified)
Air-Jet Device	19.6 ± 0.1	18.5 ± 0.3	18.9 ± 1.7
Endotracheal tube (ETT)	3.2 ± 0.9	2.1 ± 0.6	13.5 ± 0.4*
Tracheobronchial region (TB)	15.4 ± 1.9	12.7 ± 1.9	35.7 ± 4.7*
Lung chamber (LC)	49.0 ± 2.5	54.5 ± 3.3^	23.7 ± 0.3*
Filter	8.3 ± 1.4	6.6 ± 0.4	6.6 ± 0.6
Lower lung dose (LC + Filter)	57.3 ± 2.7	61.1 ± 3.0	30.3 ± 0.7*
Total lung dose (TB + LC + Filter)	72.7 ± 1.4	73.8 ± 1.2	66.0 ± 4.2*
Emitted Dose	80.4 ± 0.1	81.5 ± 0.3	81.0 ± 1.7
Total Recovery	95.6 ± 1.5	94.4 ± 2.0	98.4 ± 3.7

^Significant difference in comparison to 10 mg loaded mass; Student’s t-test, *p* < 0.05

^*^Significant difference in comparison to un-humidified condition; Student’s t-test, *p* < 0.05

Comparing 10 and 20 mg loaded doses delivered to the Rabbit LC Model with the D2 device resulted in only minor differences between the evaluated metrics. The only significant difference was in the drug mass deposited on the walls of the lung chamber, which increased from 49.0 to 54.5% of loaded dose with 20 mg mass loading. Key metrics of lower lung dose (LC + Filter) and total lung dose (TB + LC + Filter) with 20 mg loading remained high at 61.1 and 73.8%, respectively, with no significant difference from 10 mg loading. As a result, both 10 and 20 mg mass loadings with the Nano SD SLS-EEG powder appear to be good options in developing a drug delivery protocol for the *in vivo* animal model studies.

The effect of system humidification on *in vitro* rabbit lung delivery was also evaluated using a 20 mg loaded SLS-EEG formulation dose (Table V). Both lung doses were significantly different when used in the humidified system for 20 mg loaded mass delivery (*p* < 0.05) (Table V), although no differences were observed in the device retention as a result of humidification conditions. In comparison to un-humidified condition, the deposition fractions in the ETT and TB were higher, but the deposition fraction was lower in the lung chamber for humidified condition (*p* < 0.05) (Table V). Specifically, with humified conditions and a 20 mg loaded dose of the Nano SD SLS-EEG formulation, lower lung and total lung doses were reduced to 30.3 and 66.0%, respectively. While these reductions are not ideal, they do appear to be acceptable considering the degree to which large water droplets were observed to obstruct the ETT and TB flow pathways.

Overall, based on acceptable lung delivery efficiency results evaluated with realistic *in vitro* model testing, the developed formulation and device combination appeared capable of dry powder surfactant aerosol delivery via intratracheal administration for subsequent proof of concept *in vivo* testing, as described in Part II of this study. Specifically, a reasonable balance between loaded dose and aerosol performance with good lung delivery was observed when using the D2 device and 10 or 20 mg powder mass loadings of the Nano SD SLS-EEG formulation.

## Discussion

The primary premise guiding this work is that that the efficacy of AST can be increased at reduced doses, with associated reduced cost, if highly active surfactant formulations can be rapidly and effectively delivered to the lungs, beyond the lobar bronchi and preferentially all the way to the alveolar region with a relatively homogeneous deposition pattern. Most previous animal model studies of nebulized or dry powder AST have reported an administered dose of approximately 100 – 400 mg PL/kg for biological efficacy [5, 21–23, 25, 27–32]. In contrast, theoretical estimates indicate that only approximately 10 mg PL/kg delivered to the alveolar region should be required for adequate surfactant coverage of the alveoli and related surfactant function [37–40]. Our previous small-animal (rat) AST work with an animal-derived micrometer-sized Survanta-EEG formulation indicated that an approximate 1.5 mg PL/kg aerosol dose, when delivered efficiently, provided a strong physiological response, which was superior to surfactant liquid instillation [21]. In Part I of this study we have (i) presented the development of a new dry powder SLS aerosol product that may be capable of high efficacy at relatively low doses and (ii) characterized the aerosol delivery and performance of this product based on realistic *in vitro* testing in order to guide a best-case dose delivery protocol for subsequent animal model experiments. As described, the SLS aerosol product consists of a newly developed formulation (Nano SD SLS-EEG), iDP-ADS and aerosol delivery strategy. Primary observations related to each product element are reviewed below, followed by recommendations for a dose delivery protocol to test surfactant efficacy at low PL doses in surfactant-deficient animals.

Considering first the new Nano SD SLS-EEG formulation, high PL content (60%) was achieved with good powder dispersion. Our previous Survanta-EEG formulation provided approximately 27% w/w PL content with higher mass fractions of the hygroscopic excipients (40% w/w total) and similar l-leucine or trileucine (20% w/w) compared with the new SLS formulation [45, 53]. For the administration of a 30 or 60 mg total dose to an infant or test animal, it was important to reduce the water-soluble hygroscopic components, which can induce osmotic stress if not fully solubilized during aerosol transport, and now only represent 15% of the dry powder formulation by mass. L-leucine concentration was maintained at 20% w/w for particle dispersion, which should have minimal biological impact considering that it has very low water solubility and also functions as a weak surfactant [73]. Based on laser diffraction powder testing (without the air-jet DPI), high dispersion was observed with a Dv50 of 1.32 µm at 0.5 bar. The Dv50 at 4 bar better captures the expected primary particle size of 0.82 µm, as also observed in the SEM imaging. This small primary particle size together with the inclusion of the hygroscopic excipient are expected to enhance dissolution of the surfactant particles during aerosol transport and upon deposition, which is required for rapid and effective surfactant spreading and function. Furthermore, micrometer-sized primary particles deliver higher particle number concentrations to better coat the large alveolar surface area. That is, higher numbers of particles provide more deposited surface area to coat the alveolar region with a thin layer of surfactant, without increasing the mass of the delivered PL. For example, a primary particle diameter of 0.82 µm provides 6.1-fold more deposited particles than a 1.5 µm diameter particle, and 14.5-fold more deposited particles than a 2 µm diameter particle, all delivering the same total particle mass.

Considering the iDP-ADS and design of the aerosolization engine, changes from D1 to D2 devices were observed to improve the aerosol quality by doubling the FPF_<5µm_ and reducing the MMAD from approximately 2.3 to 1.7 µm. The inward protrusion of the aerosolization chamber outlet with D2 reduced the rate of aerosol release and may have retained some undispersed aggregates within the chamber; however, emitted dose was acceptable at ~ 80%. Similarly, the metal RE reduced the aerosol size increase that was previously observed to occur with the use of the GE flow diffuser [48]. A significantly larger aerosol size was emitted with the 30 mg powder mass loading. Setting the maximum MMAD at 2 µm for effective EEG aerosol delivery to an infant, both 10 and 20 mg mass loadings of the D2 device were acceptable options (i.e., had MMAD values < 2 µm). Similarly, 20 mg mass loading of the D2 design produced increased lower lung dose (LC + filter) and total lung dose (TB + LC + filter) values of ~ 61.1 and 73.8%, respectively. Delivering approximately 61% of the loaded dose beyond the upper TB model outlets and well into the lobar lung regions was impressive, especially considering the very small outlets of the upper TB model. While this lung delivery efficiency appears high relative to the large fraction of aerosol that was > 5 µm, it is noted that the iDP-ADS limited the aerosol delivery flow rate to 3 L/min, which maintained a low impaction parameter. Extreme humidification of the system with the formation of large occlusive water droplets in the TB region did significantly reduce the lower lung dose; however, 30% of the loaded dose was still delivered to this lung region.

Components of the delivery strategy that we evaluated in this study included (i) administration through an ETT interface, and (ii) delivery with positive-pressure ventilation. As described above, ETT delivery provided a majority of the aerosol to the lower lung region (in the absence of extreme humidity) and < 5% aerosol loss in the 3 mm (internal diameter) ETT. Administration of the surfactant aerosol with positive-pressure breaths is intended as a way to recruit collapsed lung regions, better distributing the aerosol to the alveoli and throughout the lungs, and then the deposited surfactant serves to hold the recruited lung regions open through dissolution and reduction of the airway surface liquid surface tension. In adult human studies with instilled surfactant liquid, failure to recruit sufficient lung regions has been presented as a reason for surfactant therapy failure [64]. Indeed, pouring a liquid into the lungs in a manner that likely pools into dependent regions would appear to be a low probability approach for opening the airways. We have limited the delivered tidal volume to the higher end of currently acceptable ventilator settings, i.e., 7 mL/kg [66, 67, 74], which is intended to be administered with the air-jet DPI for only ~ 4 to 10 breaths per aerosol delivery cycle. With this delivery strategy, we illustrated the administration of controlled lung pressure of approximately ∆P_E_ ≤ 20 cm H_2_O and LC pressure ≤ 25 ± 2 cm H_2_O. We also provided a method for maintaining these lung pressure conditions when compliance is extremely low and the tidal volume, therefore, needs to be reduced at the discretion of the clinician administering the therapy.

While not directly evaluated in this study, an important component of the aerosol delivery strategy implements the EEG approach. In the current formulation, mannitol and sodium chloride are included as hygroscopic excipients at dry powder mass fractions of 9% and 6% w/w, respectively. While this combination of hygroscopic excipients accounts for only 15% of the dry particle mass, the combination provides significant potential size increase. Based on previously developed and experimentally validated growth correlations [43, 51], the SLS-EEG particles have a growth coefficient (GC2) value of approximately 3.0, which predicts a final to initial diameter ratio increase of approximately 2.2 within the lungs at 99.5% relative humidity. As a result, particles falling within the 0.9 µm and 1.9 µm size bins will grow to 2.0 and 4.2 µm within the lungs when fully hydrated. Based on predictions of regional aerosol deposition within the lungs of infants, this size increase is expected to significantly improve the alveolar retention and reduce the exhaled dose of the aerosol [49]. Further verification of these increases in lung retention are currently underway with complete-airway computational fluid dynamics (CFD) simulations.

A primary outcome of this study is the application of the aerosol delivery characteristics to predict a dose delivery protocol for testing the efficacy of low dose AST in the rabbit surfactant washout model. As described, based on our previous animal model data for EEG formulations [21] and theoretical predictions, approximately 1.5 to 10 mg PL/kg delivered mostly to the lower lung region should be sufficient for high surfactant efficacy [37–40]. Beginning with a 60 mg total dose of the SLS-EEG formulation (administered over successive delivery cycles) and using the experimental *in vitro* delivery predictions for 20 mg individual loadings and an average expected test animal weight of 1.5 kg (consistent with both preterm infants and rabbit test-animals), the following PL dose estimates were calculated:

- Nominal Loaded PL Dose: 60 mg × 0.6 (60% PL content) = 36 mg PL
- Nominal Loaded PL/kg Dose: 36 mg PL / 1.5 kg (typical weight) = 24 mg PL / kg
- Emitted PL/kg Dose: 24 mg PL / kg × 0.8 (~ 80% emitted dose) = 19.2 mg PL / kg

Based on Rabbit LC Model testing conditions with multiple 20 mg dose loadings (and the same 60 mg total device loaded dose):

- Estimated PL / kg Lower Lung Dose: 24 mg PL / kg × 0.61 = 14.6 mg PL / kg
- Estimated PL / kg Total Lung Dose: 24 mg PL / kg × 0.74 = 17.8 mg PL / kg

Fine particle doses (FPD) of PL entering the lungs were also calculated, based on the production of the emitted PL dose and fine particle fractions, as follows:

- Fine Particle Dose < 1 µm (PL / kg): 19.2 mg PL / kg × 0.093 = 1.8 mg PL / kg
- Fine Particle Dose < 5 µm (PL / kg): 19.2 mg PL / kg × 0.271 = 5.2 mg PL / kg

As a result, with a SLS-EEG formulation dose (administered over multiple deliveries) of 60 mg, the *in vitro* lung model predicted range for lung-delivered PL / kg dose is 14.6 to 17.8 mg PL / kg. The PL FPD values that fall within the aerosol size distributions of < 5 and < 1 µm are 1.8 and 5.2 mg PL / kg, respectively. After initial deposition, some dissolution and spreading of the surfactant is expected. Some of the upper lung delivered dose will likely spread to the alveolar region over time, increasing this fraction. However, as described by Filoche *et al*. [19], there is a substantial coating cost associated with moving surfactant from the tracheobronchial to the alveolar airways, which is proportional to the size of the treated subject [19]. Furthermore, some of the aerosol may be exhaled, even with significant EEG growth. Taking all of these factors into consideration, an initial SLS-EEG formulation dose of 60 mg appears to provide a good starting point for achieving and testing the efficacy of low dose AST and approaches an initial desired range of 1.5 to 10 mg PL / kg.

In addition to the total SLS-EEG formulation dose to be administered, a primary outcome of this study is the recommended dose delivery protocol timing and use of the device, as described below. Factors to be considered in determining the dose delivery protocol included:

i. Limitation of the best-case D2 device to 10 or 20 mg powder mass loadings, partly necessitating multiple dose delivery cycles;
ii. A *dose delivery cycle* can be divided into a *aerosol delivery period* and a *stabilization and monitoring period*; the *final dose delivery cycle* does not include a *stabilization and monitoring period*;
iii. Each *dose delivery cycle* requires the test animal (or ventilated infant) to be removed from the ventilator for connection with the current D2 device at least once, causing a brief lack of PEEP support and potential risk of partial lung collapse and oxygen desaturation;
iv. The maximum amount of the SLS-EEG formulation that can be safely delivered over an *aerosol delivery period* is currently unknown; and
v. The epithelial layer is expected to be capable of absorbing and/or dissolving a majority of the hygroscopic material in the formulation within a 15–45 min window [75–77].

**Fig. 9:**
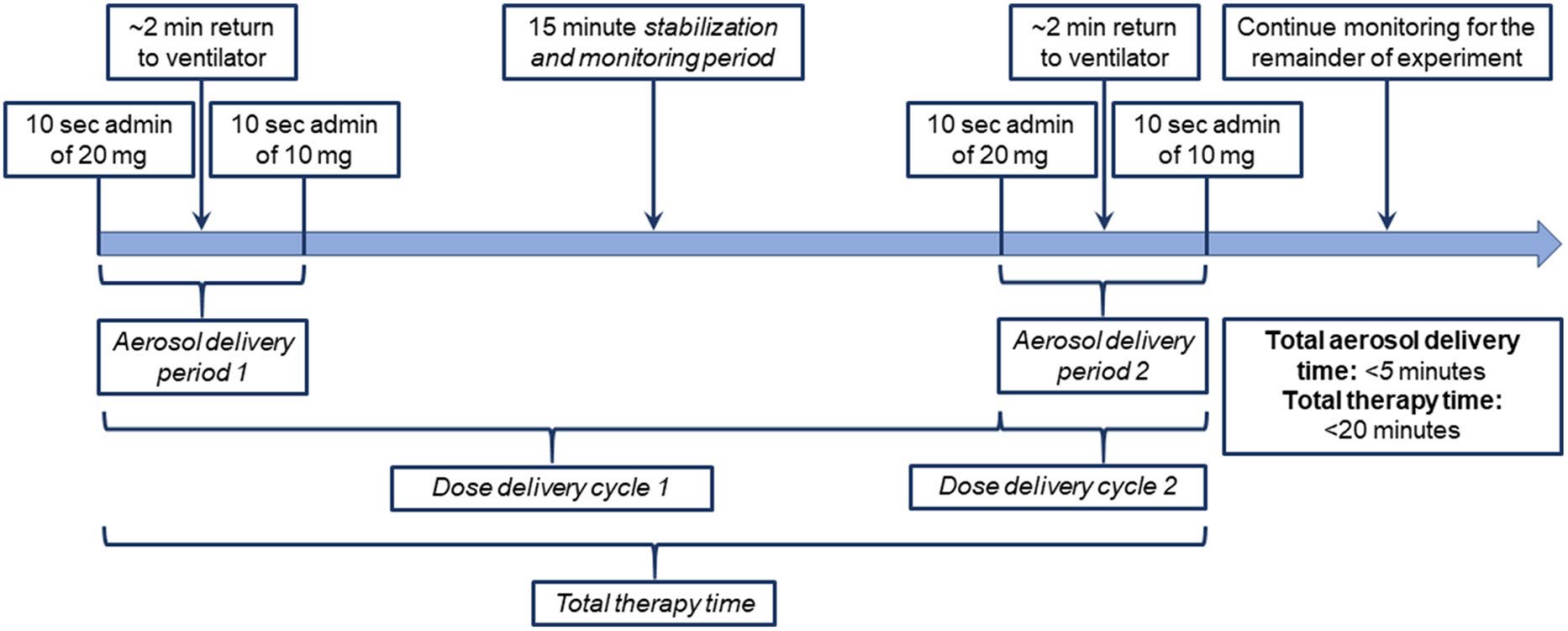
Full dose delivery protocol developed in Part I based on multiple product performance factors and biological considerations intended for *in vivo* efficacy testing in Part II.

In order to limit the amount of dry powder formulation delivered as a bolus during an *aerosol delivery period* (based on a lack of current knowledge about the SLS-EEG formulation), and to minimize the number of ventilator disconnects (which will be detrimental for oxygenation), we recommend delivering the 60 mg total dose over two equally divided 30 mg *dose delivery cycles*. Considering that only 3 mg / kg of hygroscopic material is contained within each 30 mg dose (and only 2.2 mg / kg of this reaches the lungs), we anticipate that a 15-min *stabilization and monitoring period* is more than sufficient before delivery of the second dose. The resulting full dose delivery protocol is illustrated in Fig. 9.

Based on the limitation that the D2 device can only be loaded with a maximum of 20 mg (for acceptable performance), and that aerosolization performance was moderately improved with a 10 mg loading, we recommend that each 30 mg dose be divided into subsequent 20 and 10 mg device loadings. Each of these administrations can be delivered quickly, with five actuations requiring ~ 10 s. Between these sequential 20 and 10 mg doses, the animal is required to go back on the ventilator for ~ 2 min of respiratory support, while a second device is connected to the EM Timer. As illustrated in Fig. 9, each *aerosol delivery period* therefore requires a total of ~ 2 min and 20 s (i.e., 2.3 min) based on current maximum dose loading limitations of the device.

As shown in Fig. 9, a *total therapy time* can be calculated as the sum of the *two aerosol delivery periods* and the *stabilization and monitoring period*. After the second and final *aerosol delivery period*, a subsequent *stabilization and monitoring period* is not required as part of the therapeutic delivery. Furthermore, each of the aerosol delivery periods can be assumed to require ~ 2.3 min, on average. Therefore, a *total therapy time* can be estimated to require < 20 min. Considering that a majority of this time is used for stabilization and monitoring in these early experiments, a *total aerosol delivery time* should also be considered, which can be calculated as the sum of the *aerosol delivery periods*, which is < 5 min.

During the development process, several limitations of the new aerosol product became evident that will not prevent initial animal model efficacy testing, but that should be refined in future product development work. First, considering the formulation, it is not currently clear why spray drying with the Buchi Nano system reduced the surfactant protein mimic content in the powder formulation by 50%. We did not observe any change in the B-YL concentration in the spray drying dispersion at the end of the spray drying process, indicating that any loss appears to have only affected the liquid that passed through the spray mesh and was collected as powder on the electrostatic precipitator. For content uniformity testing, seven separately produced batches of powder were considered, each sampled three times; however, future studies may need to consider expanded sampling to meet compendial standards.

Considering limitations of the device, increasing the capability for single dose loadings to 30 or 60 mg is clearly a priority. Air-jet devices capable of accepting significantly higher dose loadings and controlling the amount of powder delivered per actuation have recently been described by Howe *et al*. [46, 58]. While the administered flow rate is low and we observed good penetration through the upper TB geometry, increasing the FPF of the aerosol is also a priority.

Resistance in the Rabbit LC Model was lower than will be experienced in the animal model experiments and future nose-to-lung studies; however, compliance was accurately matched, and even with the *in vivo* studies, compliance will largely control pressure at the device and in the lungs. Furthermore, increasing the airway resistance will only increase the dissipation of pressure energy in the conducting airways and reduce pressure exposure in the alveolar region, which is where the danger of alveolar rupture and pneumothorax occur. We therefore view the recommended guidelines as conservatively safe for administering the targeted pressure ranges of 25 ± 2 and 5 ± 2 cm H_2_O. However, these guidelines will likely need to be revised and Table I updated for nose-to-lung delivery and to accommodate potential future changes in the desired positive-pressure tidal volume (e.g., 7 mL/kg) and device flow rate (e.g., currently 3 L/min).

Considering limitations of the delivery strategy, these initial animal model efficacy experiments are intended to benchmark potential efficacy. Delivery of the aerosol through the nose and to the lungs will present an additional challenge and will be addressed in future work. However, we have also made substantial progress in nose-to-lung delivery with the development of an infant air-jet DPI as reported in other studies [46–48, 58, 59]. An aerosol delivery strategy that does not require disconnection from respiratory support or that provides continuous PEEP (as with CPAP) is also needed. Finally, in determining a final dose delivery protocol, additional knowledge is needed regarding the amount of the SLS-EEG formulation that can be administered to the lungs over a single aerosol delivery period. The recommended dose delivery protocol will clearly be updated and refined pending data from the initial round of rabbit model experiments as well as with additional improvements in the device, strategy and formulation.

## Conclusions

In conclusion, a new SLS powder aerosol product was developed and refined in this study. While device powder formulation mass loadings should be limited to ≤ 20 mg, realistic *in vitro* testing indicated excellent aerosol delivery efficiency as a percentage of loaded dose to the lower lung (61.1%) and total lung (73.8%) regions. Safe positive-pressure ventilation by the device during aerosol delivery over a range of test animal weights and lung compliances was also demonstrated. Realistic *in vitro* testing in a Rabbit LC Model proved to be highly useful in developing a product that could safely deliver a majority of the aerosol to the lower lung region. With the aim of testing the efficacy of the SLS aerosol product at low surfactant doses, a dose delivery protocol was recommended that would rapidly administer two 30 mg powder masses, interspersed by a 15-min monitoring and stabilization period, equating to an approximate 20-min *total therapy administration time*. The resulting total loaded 60 mg SLS-EEG dose contains 24 mg PL / kg, which is approximately an order of magnitude below what has previously been reported in most all other animal surfactant studies, with the exception of one other EEG aerosol [21], and what is administered with the current clinical practice of high volume surfactant liquid bolus instillation.

## Data Availability

Supporting data are only available in OSF: https://osf.io/67p8h/?view_only=4ea020de08344c2597ccb857db465e97

## Abbreviations

3D: Three dimensional
ΔP_E_: Change in elastic pressure
ΔP_R_: Change in resistive pressure
AST: Aerosol surfactant therapy
C: Lung compliance
D#: Design number of air-jet DPI aerosolization engine
DPPC: Dipalmitoyl phosphatidylcholine
DPI: Dry powder inhaler
DVS: Dynamic vapor sorption
ED: Emitted dose
EM: Electromechanical
ETT: Endotracheal tube
EEG: Excipient enhanced growth
FPD: Fine particle dose
FPF: Fine particle fraction
G#: Airway generation with the trachea denoted as one
GAV: Gas actuation volume
GE: Gradual expansion
GSD: Geometric standard deviation
HPLC: High performance liquid chromatography
ID: Internal diameter
iDP-ADS: Infant dry powder aerosol delivery system
LC: Lung chamber
MMAD: Mass median aerodynamic diameter
MN: Mannitol
NaCl: Sodium chloride
NGI: Next generation impactor
NIV: Non-invasive ventilation
P_AW_: Airway pressure
P_D_: Pressure at the device (PMC unit measured)
PEEP: Positive end expiratory pressure
PIP: Peak inspiratory pressure
PL: Phospholipid
PMC: Pressure monitoring and control
POPG: 1-Palmitoyl-2-oleoyl-sn-glycero-3-phospho-(1'-rac-glycerol) (sodium salt)
Q_in_: Inhalation flow rate
R: Lung resistance
RDS: Respiratory distress syndrome
RE: Rapid expansion
RH: Relative humidity
SEM: Scanning electron microscope
SD: Spray dried or standard deviation
SLS: Synthetic lung surfactant
TB: Tracheobronchial
UV: Ultraviolent
V_t_: Tidal volume
XRPD: X-ray powder diffraction
