# Correction: Development of a New Dry Powder Aerosol Synthetic Lung Surfactant Product for Neonatal Respiratory Distress Syndrome (RDS) – Part I: *In Vitro* Testing and Characterization

**DOI:** 10.1007/s11095-024-03760-9

**Published:** 2024-09-05

**Authors:** Mohammad A. M. Momin, Dale Farkas, Michael Hindle, Felicia Hall, Robert M. DiBlasi, Worth Longest

**Affiliations:** 1 Department of Pharmaceutics, Virginia Commonwealth University, Richmond, Virginia USA; 2 Department of Mechanical and Nuclear Engineering, Virginia Commonwealth University, 401 West Main Street, P.O. Box 843015, Richmond, Virginia 23284-3015 USA; 3 Center for Respiratory Biology and Therapeutics, Seattle Children’s Research Institute, Seattle, Washington USA

**Correction: Pharmaceutical Research**

10.1007/s11095-024-03740-z

The original article has been updated to include author Robert M. DiBlasi's middle initial (incorrectly published as "Robert DiBlasi").

